# Targeting prefrontal cortex GABAergic microcircuits for the treatment of alcohol use disorder

**DOI:** 10.3389/fnsyn.2022.936911

**Published:** 2022-08-29

**Authors:** Kenneth N. Fish, Max E. Joffe

**Affiliations:** Translational Neuroscience Program, Department of Psychiatry, University of Pittsburgh, Pittsburgh, PA, United States

**Keywords:** electrophysiology, addiction, drug abuse, preclinical models, interneurons, synaptic plasticity, GABA, glutamate

## Abstract

Developing novel treatments for alcohol use disorders (AUDs) is of paramount importance for improving patient outcomes and alleviating the suffering related to the disease. A better understanding of the molecular and neurocircuit mechanisms through which alcohol alters brain function will be instrumental in the rational development of new efficacious treatments. Clinical studies have consistently associated the prefrontal cortex (PFC) function with symptoms of AUDs. Population-level analyses have linked the PFC structure and function with heavy drinking and/or AUD diagnosis. Thus, targeting specific PFC cell types and neural circuits holds promise for the development of new treatments. Here, we overview the tremendous diversity in the form and function of inhibitory neuron subtypes within PFC and describe their therapeutic potential. We then summarize AUD population genetics studies, clinical neurophysiology findings, and translational neuroscience discoveries. This study collectively suggests that changes in fast transmission through PFC inhibitory microcircuits are a central component of the neurobiological effects of ethanol and the core symptoms of AUDs. Finally, we submit that there is a significant and timely need to examine sex as a biological variable and human postmortem brain tissue to maximize the efforts in translating findings to new clinical treatments.

## Background

Alcohol use disorders (AUDs) present a tremendous burden on individuals suffering from the disease, their immediate and extended families, and society at large (Hasin et al., 2007; Whiteford et al., 2013; Grant et al., 2017). The United States Food and Drug Administration has approved disulfiram, naloxone, and acamprosate for AUD; however, these options are limited by insufficient efficacy, unsatisfactory side effects, and relatively low compliance (Jonas et al., 2014). Developing novel treatments is, therefore, of paramount importance for improving patient outcomes. Rational treatment development, in turn, will be driven by a better understanding of the molecular and neurocircuit mechanisms through which ethanol exposure alters brain function. This review summarizes historical findings and suggests that changes in fast transmission through the frontal cortex inhibitory microcircuits are a central component of ethanol's neurobiological effects. In addition, we highlight recent mechanistic studies in preclinical models that provide new avenues for breakthrough treatment approaches and argue there is a tremendous need to examine sex as a biological variable and a human postmortem brain tissue to maximize efforts in translating findings to new clinical treatments.

## Prefrontal cortex inhibitory neurons as specific and selective targets

Excessive craving and motivation to drink, along with challenges in moderating consumption are key symptoms associated with AUDs. Clinical studies have consistently associated the prefrontal cortex (PFC) function with these behaviors and symptoms (Grusser et al., 2004; Koob, 2014; Courtney et al., 2016; Blaine et al., 2020; Yang et al., 2021). In addition, population-level analyses have linked the PFC structure and function with heavy drinking and/or AUD diagnosis (Medina et al., 2008; Sorg et al., 2012; Wang et al., 2016). Finally, several interventional studies have linked a direct stimulation of PFC areas with reductions in alcohol craving and drinking metrics (Boggio et al., 2008; Addolorato et al., 2017; Philip et al., 2020), providing exciting proof-of-concept for the potential of new tools to modulate PFC function as breakthrough treatments for AUDs. Together, these translational and clinical studies motivate continued basic neuroscience studies and postmortem studies to identify PFC cell populations that may be modulated as novel standalone or adjunct treatments for AUDs.

**Figure 1:**
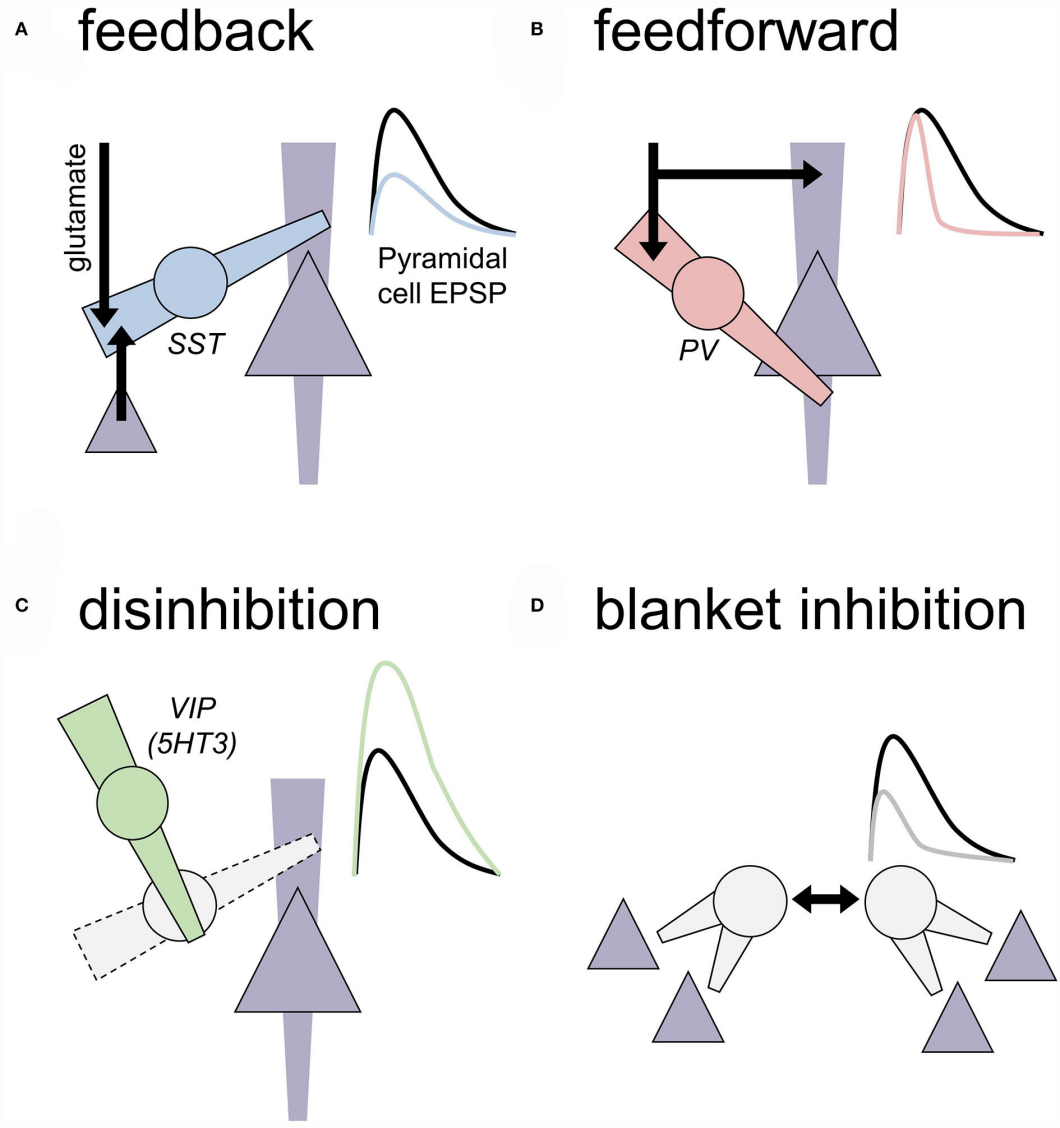
Motifs of local inhibition in the central nervous system. Generalized motifs of cortical inhibition are depicted that include cellular/subcellular targets and approximated effects on an excitatory postsynaptic potential (EPSP) recorded from a nearby pyramidal cell. To some extent, these inhibitory transmission motifs are present across all cortical layers, but some motifs are more prevalent in some layers based on where IN subtypes reside. **(A)** Feedback inhibition. A local inhibitory neuron (IN; circle) receives an excitatory transmission from long-range glutamate inputs or local competing ensembles or cortical columns (black arrows). Feedback INs regulate dendritic compartments of principal neurons (triangle) to decrease the amplitude of EPSPs. Cortical somatostatin (SST) INs represent a primary example of feedback INs. **(B)** Feedforward inhibition. A local IN and neighboring principal neuron receive coincidental excitatory input. Feedforward INs, often expressing parvalbumin (PV), are optimized to inhibit the nearby cell bodies rapidly and faithfully after receiving excitatory inputs. Through this process, feedforward INs restrict the window during which EPSPs may be converted into action potential firing. Feedforward INs can regulate dozens of related principal cells to recruit neuronal ensembles and generate oscillatory activity in many brain areas. **(C)** Disinhibition. INs can inhibit other INs to relieve inhibition and ultimately facilitate EPSPs on the principal neurons. Prime examples of this motif include SST-INs inhibiting PV-INs and vasoactive intestinal peptide (VIP) INs inhibiting SST-INs. **(D)** Blanket inhibition. Some INs subtypes help drive their activity to maintain wide networks of inhibition and low levels of excitatory drive throughout broad brain areas. For example, PV-INs form extensive gap junctions with other PV-INs and cholecystokinin INs can co-release glutamate to excite onto each other.

Several properties make gamma-aminobutyric acid (GABA) inhibitory neurons (INs) highly attractive targets for developing novel pharmacological treatments for AUD and other neuropsychiatric diseases (Box 1). In human PFC, the INs make up approximately 20% of all neurons. There are more than 20 IN subtypes, each displaying distinct transcriptional programs (Tasic et al., 2016, 2018; Tremblay et al., 2016; Krienen et al., 2020). Thus, it may be possible to specifically modulate transcriptionally unique IN subtypes without altering other INs or cell types, providing opportunities to develop treatment mechanisms with a low risk of deleterious side effects. Moreover, the tremendous diversity in the form and function of IN subtypes (Figure 1) confers them with a great potential for developing means to restore specific pathophysiological disruptions.

Box 1 **Assembly and function of GABA_A_ receptors.**

GABA_A_ receptors are ligand-gated anion channels that generally hyperpolarize neurons by fluxing chloride ions (Mody and Pearce, 2004; Lobo and Harris, 2008). GABA_A_ receptors are members of the Cys-loop superfamily and assemble as obligate pentamers. Several GABA_A_ receptor subunits exist, generating a wide variety of combinations of functional channels in the mammalian CNS. There are six types of α subunits, three β, three γ, and one δ, ε, π, and θ. There are also three ρ subunits, but these generally assemble homopentamers and are classified as GABA_C_ receptors. The most abundant GABA_A_ receptor assemblies contain two α, two β, and one γ subunit. GABA binds on the interface between α and β subunits facilitating a structural rearrangement that allows for chloride ions to permeate the central pore.

The combinatorial nature of GABA_A_ receptor assembly yields a great diversity in function. The subunit stoichiometry of a specific GABA_A_ pentamer can affect its subcellular localization and biophysical properties, such as GABA affinity, desensitization/deactivation kinetics, and voltage rectification (Mody and Pearce, 2004; Olsen and Sieghart, 2009; Lee and Maguire, 2014). From this inherent diversity, GABA_A_ subunits have garnered significant attention in translational studies aimed at developing selective approaches to modulate inhibitory circuit function. For example, α1-containing GABA_A_ receptors regulate the sedative and amnestic effects of benzodiazepines, whereas their anxiolytic properties are mediated by alternative α isoforms (Rudolph et al., 1999; Kralic et al., 2002). Thus, subunit-selective GABA_A_ receptor modulators have the potential to preserve the therapeutic effects of existing medications while mitigating undesirable side effects (Mohler, 2006).

GABA_A_ receptor subunit isoforms display heterogeneous patterns of expression. α1, β1-3, and γ2 GABA_A_ subunits are expressed widely throughout the brain (Wisden and Seeburg, 1992; Pirker et al., 2000; Hortnagl et al., 2013). α3-5, γ1, γ3, and δ subunit isoforms, in contrast, are expressed in distinct, restricted patterns throughout the forebrain. α6 is essentially restricted to the cerebellum. Genetic deletion experiments suggest that γ2 subunits are absolutely critical for broad synaptic inhibitory transmission (Gunther et al., 1995; Martenson et al., 2017), whereas the more abundant α or β isoforms are each dispensable or redundant to some extent (Homanics et al., 1997; Sur et al., 2001; Vicini et al., 2001). Synaptic receptors mediate phasic transmission, whereas certain GABA_A_ receptor subunit combinations are enriched at extrasynaptic sites and facilitate tonic inhibitory currents (Belelli et al., 2009; Lee and Maguire, 2014). These extrasynaptic receptors commonly assemble with α5, γ2, and/or δ subunits, conferring them with a high affinity for GABA and slow desensitization, providing prolonged inhibition in response to low concentrations of GABA. Extrasynaptic receptors have been the subject of recent reviews covering their basic neurobiology (Lee and Maguire, 2014) as well as more focused analyses with respect to AUD (Weiner and Valenzuela, 2006; Lovinger and Homanics, 2007).

Even among synaptic GABA_A_ receptors, the subunit composition has a major effect on subcellular localization. While α1 subunits are found in synapses across all subcellular domains (Nusser et al., 1996), other isoforms display restricted subcellular expression patterns. α5 subunits, for example, are primarily localized to inhibitory synapses in pyramidal cell dendrites (Magnin et al., 2019), where they receive GABA from SST-INs but not PV-INs (Schulz et al., 2018). In contrast, α2 subunits are expressed at higher levels postsynaptic to chandelier IN inputs onto the axon initial segment (Nusser et al., 1996) and in somatic synapses that preferentially receive inputs from CCK-INs (Nyiri et al., 2001). Together, these findings raise the possibility that subunit-selective GABA_A_ receptor allosteric modulators could be developed to selectively modulate distinct microcircuits.

Adaptations in PFC inhibitory microcircuits are associated with changes in mood, decision-making, executive control, and symptom domains that are present in some individuals with an AUD. Interestingly, disruptions to inhibitory microcircuits have been observed in several comorbid brain diseases including affective disorders and schizophrenia (Lewis et al., 2005, 2012; Luscher et al., 2011; Fogaca and Duman, 2019), supporting the idea that IN pathophysiology may be a feature of AUDs as well. Nonetheless, our understanding of AUD-associated microcircuit disruptions in the human brain lags substantially behind that of other psychiatric diseases, and warrants postmortem studies with AUD patient population samples.

Mechanistic research focused on the PFC has gained traction since the early 1990's (Laubach et al., 2018) after it was shown that the primate dorsolateral prefrontal cortex (dlPFC) (Brodmann's areas (BA)9 and BA46) was a working memory hub (Funahashi et al., 1989; Williams and Goldman-Rakic, 1995). The findings from these studies were supported by those of contemporaneous human neuroimaging studies that implicated the dlPFC in higher-order cognitive processing, including working memory (Pardo et al., 1991; McCarthy et al., 1994). Although the rodent PFC was initially believed to be anatomically and functionally similar to the primate dlPFC, a substantial amount of data challenges this view (extensively reviewed in Laubach et al., 2018). Especially in mice, which do not have a granular layer 4 within the PFC, there is no equivalent to the primate BA9 and BA46, which is where most “PFC” studies have focused. In rodents, the focus is frequently on the medial PFC and its three main subregions: cingulate, prelimbic, and infralimbic. While these subregions are involved in working memory processes, affective behaviors, and motivation, the degree to which rodent PFC regions map onto primate and human homologs remain an area of significant debate.

## Non-overlapping neocortical IN subpopulations

In general, INs within the rodent neocortex can be split into three non-overlapping classes. INs that arise from the medial ganglionic eminence express either the Ca^2+^-binding protein parvalbumin (PV) or the neuropeptide somatostatin (SST), whereas INs arising from the caudal ganglionic eminence (CGE) express the ionotropic serotonin receptor, 5HT3a subtype (Lee et al., 2010; Pfeffer et al., 2013; Tremblay et al., 2016; Lim et al., 2018). For the most part, these three major classes exist in humans. There are, however, important differences in the proportion of GABA neurons immunoreactive for subtype-specific markers between rodents and primates. In primates, approximately 50% of GABA neurons are calretinin (CR) immunoreactive, while approximately 20% are PV immunoreactive (Conde et al., 1994; Gabbott and Bacon, 1996; Krienen et al., 2020). In contrast to primates, CR neurons are not considered a canonical GABA subtype in rodents; instead, vasoactive intestinal peptide (VIP), which highly colocalizes with CR in primates, is frequently used to define a major group of INs in rodents. Approximately, 20% of GABA neurons in rodents are VIP positive, while nearly half are PV immunoreactive. Considering these IN subtypes that integrate within PFC microcircuits in distinct manners (refer to next section for details), this difference is expected to differentially impact the local and global network. Here, we provide an overview of each of these three major classes of INs based on rodent studies, pointing out important species differences where relevant, to orient the reader and aid in interpreting functional findings from the clinic.

### Parvalbumin INs

In the cortex, the PV is mainly expressed in two morphologically unique GABAergic neurons, PV basket cells and PV chandelier cells (Celio, 1986; Kawaguchi et al., 1987; Kosaka et al., 1987; DeFelipe, 1993; Hu et al., 2014). Approximately, 80% of PV-INs are PV basket cells. In the rodent neocortex, PV basket cells mainly reside in the middle layers while most PV chandelier cells are located along the layer 1–2 boarder and in deep layers. PV-INs have been studied more extensively than other IN classes, in part due to their distinguishing firing properties including high firing frequency, rapid action potential kinetics, and minimal spike-firing adaptation (McCormick et al., 1985; Connors and Gutnick, 1990; Kawaguchi, 1993a,b; Kawaguchi and Kubota, 1997, 1998; Markram et al., 2004; Rainnie et al., 2006; Woodruff and Sah, 2007; Gittis et al., 2010; McGarry and Carter, 2016; O'Hare et al., 2017; Joffe et al., 2020; Unal et al., 2020). These distinctive intrinsic properties provided opportunities to examine PV-IN function nearly two decades before the advent of genetic tools to identify and modulate specific IN subpopulations. Throughout this review, the term, “PV-IN” will be used broadly when referring to PV basket cells including experiments that only classified neurons by their firing properties.

Several properties of PV-INs confide them with an ability to synchronize neural ensembles, ultimately coupling and strengthening the network activity (Cobb et al., 1995; Royer et al., 2012; Knoblich et al., 2019). PV-INs mainly innervate the perisomatic region (basket cells— proximal dendrites and soma; chandelier cells —axon initial segment) of principal neurons and other PV-INs (Hu et al., 2014). The perisomatic innervation and rapid signal propagation kinetics provide PV-INs with the ability to efficiently transmit feed-forward inhibition (Mallet et al., 2005) (Figure 1B). Approximately, half of the synapses onto PV-INs arise from local axon collaterals (Melchitzky and Lewis, 2003); the other half comes from long-range excitatory afferents, with only approximately 2% from the thalamus (Rotaru et al., 2005). Relative to principal cells, PV-IN dendrites display rapid, high-conductance excitatory postsynaptic currents (EPSCs) related to high glutamate release probability, GluA1/GluA4-containing α-amino-3-hydroxy-5-methyl-4-isoxazolepropionic acid (AMPA) receptors, and transmembrane AMPA receptor regulatory proteins (Box 2) (Geiger et al., 1995; Gittis et al., 2010, 2011; Tao et al., 2013; Lalanne et al., 2016). Uncommon assemblies of AMPA receptor subunits (Bowie and Mayer, 1995; Geiger et al., 1995) confer PV-INs with distinct synaptic plasticity rules and mechanisms (Sambandan et al., 2010; Szabo et al., 2012; Manz et al., 2020). In contrast, PV-INs display minimal postsynaptic NMDA receptor currents (Matta et al., 2013; McGarry and Carter, 2016; Bogart and O'Donnell, 2018), although presynaptic NMDA receptors do regulate PV-IN GABA release (Pafundo et al., 2018). At the PV-IN cell body, voltage-gated Kv3.1 potassium channels contribute to rapid and non-accommodating spiking (Chow et al., 1999; Rudy and McBain, 2001; Goldberg et al., 2005), which rarely fails and efficiently propagates electrical signals down the PV-IN axon (Doischer et al., 2008). Throughout the axon, the expression of the sodium/potassium ATPase (Anderson et al., 2010), hyperpolarization-activated cation channels (Roth and Hu, 2020), and other sodium channels (Hu et al., 2018) counters the metabolic demands of high frequency firing. At PV-IN presynaptic terminals, synaptotagmin 2 (Sommeijer and Levelt, 2012; Bouhours et al., 2017) and a small number of ω-conotoxin-sensitive P/Q type voltage-gated Ca^2+^ channels (Zaitsev et al., 2007; Bucurenciu et al., 2010; Rossignol et al., 2013) contribute to high-fidelity GABA release from the top of 10 release sites (Buhl et al., 1994; Kraushaar and Jonas, 2000), generating GABAergic inhibitory events with the rapid rise and decay kinetics (Gittis et al., 2010; Marlin and Carter, 2014; Unal et al., 2020). Thus, through specialized molecules in several subcellular compartments, PV-INs transmit feedforward inhibition of coincidental principal cell excitatory postsynaptic potentials within 5–10 ms, facilitating spike-timing-dependent synaptic plasticity (Tremblay et al., 2016).

Box 2 **Ionotropic glutamate receptor expression in INs.**

Glutamate mediates fast excitatory transmission *via* cationotropic cell surface receptors (Mayer and Armstrong, 2004; Traynelis et al., 2010; Paoletti et al., 2013). Glutamate receptors assemble as obligate tetramers and can be split into three main types, AMPA receptors, kainate receptors, and NMDA receptors. For all three types, selective cationic pores form within the center of tetrameric assemblies and open when glutamate binds within large extracellular domains. AMPA and kainate receptors flux Na^+^ to depolarize membranes from the rest. In contrast, NMDA receptors do not effectively transmit currents at hyperpolarized membrane potentials but allow for Na^+^ and Ca^2+^ to enter during mild depolarization. Through these properties, NMDA receptors readily detect coincidental synaptic transmission and promote Ca^2+^-dependent plasticity.

The four AMPA receptor subunits of GluA1-4 exhibit slight differences in biophysical properties (Mayer and Armstrong, 2004; Traynelis et al., 2010). The RNA editing of the *Gria2* transcript alters a key peptide residue in the GluA2 pore-forming region such that GluA2-containing AMPA receptors are selectively permeable to monovalent cations (Burnashev et al., 1992). Of note, AMPA receptors that lack GluA2 not only flux monovalent cations but also allow for Ca^2+^ ions to pass (Hollmann et al., 1991). AMPA receptors that contain GluA2 subunits are the most common assemblies in principal neuron synapses, whereas many types of inhibitory INs express AMPA receptors that lack GluA2. Variation in channel opening kinetics, glutamate affinity, and interactions with auxiliary proteins and intracellular polyamines also confer unique properties to distinct assemblies of AMPA receptor subunits enriched on INs (Soto et al., 2007; Ziff, 2007). Most types of INs also express kainate receptors, primarily those containing GluK1 and GluK2 subunits (Cauli et al., 2000; Pelkey et al., 2017; Huntley et al., 2020). Pre and postsynaptic kainate receptors regulate IN excitability, inhibitory transmission onto principal neurons, and network oscillations (Cossart et al., 1998; Frerking et al., 1998; Fisahn et al., 2004; Daw et al., 2010).

NMDA receptors must contain two GluN1 subunits that bind glycine or D-serine, and generally include two GluN2 subunits (Paoletti et al., 2013; Iacobucci and Popescu, 2017). The glutamate interacts with the GluN2 subunits, which exist in four isoforms produced from four genes (GluN2A-D, *Grin2A-D*). GluN2A and GluN2B are the most abundant. GluN2B-containing NMDA receptors display longer channel opening kinetics and a higher affinity for glutamate than their GluN2A-containing counterparts. These properties enhance channel conductance and make GluN2B an important signaling molecule under conditions with low glutamate concentrations, such as extrasynaptic domains and in nascent, silent synapses particularly important for long-term plasticity and adaptive responses to drugs of abuse (Kerchner and Nicoll, 2008; Dong, 2016). GluN2C and GluN2D subunits have even higher affinities for glutamate, slower desensitization kinetics, and transmit more current at hyperpolarized potentials (Paoletti et al., 2013; Iacobucci and Popescu, 2017). GluN2C and GluN2D display relatively restricted patterns of expression in adulthood, but these receptor subunits are notably enriched in cortical INs (Monyer et al., 1994; Perszyk et al., 2016; Hanson et al., 2019; Garst-Orozco et al., 2020).

NMDA receptor inhibition is an important component underlying some of the physiological effects of ethanol (Woodward, 2000; Lovinger and Roberto, 2013). Relative to other ionotropic receptors, ethanol preferentially inhibits charge transfer through NMDA receptors (Lovinger et al., 1989, 1990; Morrisett et al., 1991; Yaka et al., 2003; Hendricson et al., 2004), and potencies of alcohol for inhibiting NMDA receptors and intoxicating humans(Lovinger et al., 1989, 1990). More recent molecular studies have revealed that mutations in the modulatory extracellular N-terminal domain and transmembrane regions of multiple NMDA receptor subunits alter the inhibitory actions of ethanol (Ronald et al., 2001; Woodward and Smothers, 2003; Smothers et al., 2013; Smothers and Woodward, 2016). Specific mutations within the GluN1 transmembrane region also alter several behavioral effects of low-dose ethanol (den Hartog et al., 2013). Taken together, several lines of molecular, circuit-level, and behavioral studies suggest that direct interactions with NMDA receptors regulate some of the central effects of ethanol.

In addition to their specialized cellular properties, PV-INs are integrated at the circuit-level to promote concerted network activity. PV-INs express gap junction proteins, and a single PV-IN may be electrically connected with over 60 others (Katsumaru et al., 1988; Gibson et al., 1999; Fukuda and Kosaka, 2000, 2003; Muller et al., 2005; Fukuda et al., 2006; Woodruff and Sah, 2007). PV-INs also display extensive reciprocal GABAergic synapses that can readily undergo LTP (Galarreta and Hestrin, 2002; Sarihi et al., 2012; Pfeffer et al., 2013). PV-INs serve a key role in regulating γ oscillations, a high frequency range whose power increases during complex tasks that require high cognitive demand (Bartos et al., 2007; Gonzalez-Burgos et al., 2015). The PV-IN activity tracks closely to γ oscillations (Tukker et al., 2007), and PV-IN manipulations bidirectionally regulate the power of γ oscillations (Cardin et al., 2009; Sohal et al., 2009; Chen et al., 2017; Cho et al., 2020). Taken together, the molecular, cellular, and microcircuit-level properties establish PV-INs as key actuators controlling the gain within locally assembled neural networks.

### Somatostatin INs

In the neocortex, SST-expressing neurons are exclusively GABAergic (Hendry et al., 1984a; Melchitzky and Lewis, 2008; Urban-Ciecko and Barth, 2016). Most SST-INs are low-threshold spiking cells, characterized by high input resistance, depolarized resting potential, low rheobase, and occasional firing upon hyperpolarization rebound (Kawaguchi and Kubota, 1997, 1998; Marlin and Carter, 2014; McGarry and Carter, 2016; Tremblay et al., 2016; Nigro et al., 2018; Joffe et al., 2020; Unal et al., 2020). The vast majority of SST-IN synaptic contacts are made with dendrites of non-GABAergic neurons (Katona et al., 1999; Muller et al., 2007; Melchitzky and Lewis, 2008). For example, a morphologically unique SST-IN, the Martinotti cell, primarily synapses on the most distal dendrites (in layer 1) of nearby pyramidal cells, providing feedback inhibition between competing ensembles or cortical columns (de Lima and Morrison, 1989; Kawaguchi and Kubota, 1997; Muller et al., 2007; Murayama et al., 2009; Marlin and Carter, 2014; Unal et al., 2020) (Figure 1A). Through their synapses onto pyramidal cell dendrites, SST-INs regulate Ca^2+^ signaling and NMDA receptor activation (Gentet et al., 2012; Lovett-Barron et al., 2012; Chiu et al., 2013; Marlin and Carter, 2014; Ali et al., 2020), and, conversely, NMDA receptor activation can strengthen SST-IN inputs to pyramidal cells through heterosynaptic LTP of GABA_A_ transmission (Chiu et al., 2013; Horn and Nicoll, 2018). Furthermore, SST-IN synapses on principal cells are enriched with α5-containing GABA_A_ receptors, which display outward rectification profiles comparable to NMDA receptors (Schulz et al., 2018). These α5-containing receptor currents provide shunting inhibition to attenuate NMDA receptor function, action potential back propagation, and spike-timing-dependent plasticity (Groen et al., 2014; Schulz et al., 2018). Together, the tight coordination with NMDA receptor signaling suggests that SST-INs are highly specialized for regulating plasticity at active, depolarized synapses while mitigating the liability for broad blanket inhibition. Consistent with these molecular studies, the SST-IN regulation of principal cell dendrites is critical for spine reorganization to support skill acquisition (Chen et al., 2015) and rapid antidepressant response (Ali et al., 2020). The preclinical literature broadly supports the idea that SST-INs are key substrates for filtering and guiding experience-dependent synaptic plasticity.

Somatostatin-inhibitory neurons are uniquely integrated into local circuits relative to other types of INs. Unlike the depressing transmission observed onto most types of INs, SST-INs display strongly facilitating excitatory input (Pouille and Scanziani, 2004; Silberberg and Markram, 2007; Xu et al., 2013; McGarry and Carter, 2016). The characteristic low probability of glutamate release onto SST-INs is guided in *trans* by the postsynaptic expression of extracellular leucine-rich repeat fibronectin containing 1 (Elfn1) protein (Sylwestrak and Ghosh, 2012). Transsynaptic modulation by Elfn1 subsequently promotes the constitutive activity of presynaptic mGlu_7_ receptors, thereby maintaining low glutamate release probability onto SST-INs (Shigemoto et al., 1996; Dunn et al., 2018; Stachniak et al., 2019). These facilitating and summating excitatory synapses, in combination with a high membrane resistance, allow SST-INs to respond to repeated stimulation from even just one presynaptic neuron (Kapfer et al., 2007; Silberberg and Markram, 2007). This “winner-take-all” cellular logic has been proposed as a primary means through which competing ensembles influence and filter each other (Tremblay et al., 2016). Accordingly, SST-INs inhibit pyramidal cells during high frequency barrage, while the influence of other IN subtypes wanes due to their depressing synapses (Beierlein et al., 2003; Gonzalez-Burgos et al., 2004; Kapfer et al., 2007; McGarry and Carter, 2016). High frequency stimulation can also induce the release of SST peptide (Dao et al., 2019), which can modulate microcircuit function through 5 subtypes of G protein-coupled receptors (Viollet et al., 2008; Yavorska and Wehr, 2016). In addition to their specialized responses to sustained stimulation, the spontaneous SST-IN activity *in vivo* can regulate pyramidal cells through metabotropic GABA_B_ receptors (Gentet et al., 2012; Urban-Ciecko et al., 2015; Urban-Ciecko and Barth, 2016). Furthermore, the molecular logic guiding synaptic plasticity onto SST-INs contrasts sharply with that in pyramidal cells. SST-INs display anti-Hebbian long-term potentiation, whereby postsynaptic hyperpolarization can increase EPSCs in response to tetanic stimulation (Lamsa et al., 2007). The long-term potentiation (LTP) of SST-IN is induced through postsynaptic CP-AMPA receptors and mGlu_1_/mGlu_5_ receptors, although maintenance may occur at presynaptic sites (Oren et al., 2009; Le Duigou and Kullmann, 2011; Maksymetz et al., 2021; Joffe et al., 2022). Alterative splicing of *Grm1* and several AMPA receptor genes may contribute to the distinct synaptic plasticity mechanisms that occur in SST-INs (Furlanis et al., 2019). Collectively, these findings suggest that plastic interactions between SST-INs and their local networks are critical for long-term changes in how microcircuits filter incoming information.

In addition to Martinotti cells, several other GABAergic subtypes express SST in the neocortex and hippocampus (Oliva et al., 2000; Ma et al., 2006; Hu et al., 2013; Mikulovic et al., 2015; Yavorska and Wehr, 2016). In fact, Yavorska and Wehr estimated that neocortical SST-INs can be further subdivided by form, function, and gene expression into potentially 100 subgroups (Yavorska and Wehr, 2016). In addition to morphological and physiological studies, the SST-IN subdivision has been motivated by the serendipitous, restricted expression of GFP in specific GABAergic cell types (Oliva et al., 2000; Ma et al., 2006). One GAD67-GFP line, termed X98, strictly labels CB-expressing Martinotti cells, while another line, X94, can be used to identify stuttering INs in layer 4 (Ma et al., 2006). Later studies discovered that SST-INs labeled in the X94 line, in stark contrast to Martinotti cells, project only within deep layers of the cortex and preferentially target PV-INs (Xu et al., 2013). While the rodent frontal cortex does not contain a layer 4 *per se*, similar SST-IN disinhibitory motifs have been proposed to exist there (Xu et al., 2019; Cummings and Clem, 2020), in the motor cortex (Zhang et al., 2016), and the visual cortex (Pfeffer et al., 2013). Furthermore, supporting the existence of distinct SST-IN subpopulations, comparative studies of genetically-defined INs often discover that SST-INs functionally segregate into at least two subtypes (Kvitsiani et al., 2013; Knoblich et al., 2019). Finally, several studies have shown that a relatively small number of SST-INs, primarily located in layer 6, co-express neuropeptide Y (NPY), neuronal nitric oxide synthase (nNOS), and the substance P receptor 1 (Tomioka et al., 2005), and send long-range inhibitory projections to the striatum and other extracortical areas (Rock et al., 2016).

### 5HT3-INs

The final major class of neocortical INs arises from the CGE. In rodents, but not primates, nearly all CGE-derived INs express the 5HT3a serotonin receptor (Lee et al., 2010; Krienen et al., 2020). 5HT3-INs are primarily localized within the supragranular layers of the neocortex and potentially represent the most diverse of the three non-overlapping classes of INs. Based on morphology, cells in this class have been described as basket cells, bipolar cells, multipolar cells, single (and some double) bouquet cells, and neurogliaform cells (Lim et al., 2018). It is important to note that in primates, some of these morphological features are shared with or restricted to other IN classes. For example, in primates, CR expression is often used as a broad classifier of CGE-derived neurons, and approximately 25% of CR-IN synapses target pyramidal neurons (Melchitzky and Lewis, 2008). Coordinated translational research spanning rodent and primate models will be important for understanding and reconciling species differences in CGE-derived IN physiology.

From a molecular standpoint, 5HT3-INs can be divided into two groups in the rodent neocortex, each containing transcriptionally unique cells, based on the expression of VIP) (Schuman et al., 2019). VIP-expressing INs are the largest and best-studied subgroup, comprising about 40% of INs in the superficial neocortex (Lee et al., 2010). VIP-INs can be split into two, generally, non-overlapping subpopulations based on the expression of cholecystokinin (CCK) or CR (Porter et al., 1998; He et al., 2016; Tasic et al., 2018). VIP/CCK-INs are basket cells closely related to other CCK-INs (Tasic et al., 2018). In contrast, VIP/CR-INs are bipolar cells whose terminals avoid pyramidal cells and instead target other IN subtypes, especially SST-INs (Lee et al., 2013; Pfeffer et al., 2013; Pi et al., 2013; Fu et al., 2015; Anastasiades et al., 2018) (Figure 1C).

Most 5HT3-INs that do not express VIP express LAMP5 (Tasic et al., 2016, 2018), a brain-specific membrane protein that regulates presynaptic neurotransmission (Tiveron et al., 2016). Interestingly, deep-layer LAMP5-INs are approximately 10-fold more abundant in primates than in rodents (Krienen et al., 2020), but their functional significance is unknown. 5HT3-INs that do not express VIP can be further subdivided into three non-overlapping subtypes [NDNF/NPY+, NDNF/NPY-, or α7 nACh receptor+], each with a characteristic electrophysiological signature (Schuman et al., 2019).

Glutamate synapses onto 5HT3-INs tend to exhibit large EPSCs from NMDA receptors and GluA2-containing CI (calcium-impermeable)-AMPA receptors (Szabo et al., 2012; Matta et al., 2013). With the important exception of CCK-INs, monosynaptic inputs from 5HT3-INs to pyramidal cells are biased toward apical dendrites even more than SST-INs (Marlin and Carter, 2014). In addition, a major function of 5HT3-INs is to relieve inhibition of principal neurons by quietening other IN subtypes (Tremblay et al., 2016). Many subtypes of 5HT3-INs are also characterized by high co-expression of neuropeptides (Vincent et al., 1982; Hendry et al., 1984a,b). The signaling and functions of VIP (White et al., 2010; Harmar et al., 2012), CCK (Rotzinger and Vaccarino, 2003), NPY (Robinson and Thiele, 2017; Thorsell and Mathe, 2017), and corticotropin-releasing factor (CRF) (Agoglia and Herman, 2018) peptides are complex, have been reviewed well, and will not be discussed here in detail. Instead, this review treats these neuropeptides as cell type-specific markers and describes how IN subclasses are specialized to regulate principal neurons through GABA transmission.

Electrophysiological studies in rodents have found that VIP-INs are functionally distinguished by high input resistance and adaptive action potentials (Lee et al., 2013; Marlin and Carter, 2014; Pronneke et al., 2015; Anastasiades et al., 2018; Schuman et al., 2019; Dudai et al., 2020). On the other hand, CCK-IN basket cells fire in a regular-spiking pattern, display long dendritic Ca^2+^ transients and release GABA in a bulky asynchronous manner (Kawaguchi and Kubota, 1998; Bartos and Elgueta, 2012; Tremblay et al., 2016). Neocortical CCK-INs transmit short-latency inhibitory postsynaptic currents that broadly inhibit multiple types of neurons, including pyramidal cells and other classes of INs (Nguyen et al., 2020). CCK-INs receive weak inhibition and depressing excitatory transmission, which, along with a long membrane time constant, allow for CCK-INs to summate information from separate afferents (Glickfeld and Scanziani, 2006). Therefore, like PV-INs, CCK-INs may detect and augment coincidental information to regulate the gain within local neural microcircuits.

## Evidence from humans linking GABAergic circuit function with AUDs

### Genetic studies

Many studies have detected relationships between AUD diagnosis and variation in genes involved with GABA_A_ receptor function (Table 1). Over 20 studies have associated single nucleotide polymorphisms (SNPs) within the α2 GABA_A_ receptor subunit gene, *GABRA2*, and either AUD diagnosis or the acute effects of ethanol (Covault et al., 2004; Edenberg et al., 2004; Haughey et al., 2008; Roh et al., 2011; Yang et al., 2017; Koulentaki and Kouroumalis, 2018). Consistent with these studies, distinct ethanol-related phenotypes have been observed in mice with genetically-altered ethanol-insensitive α2 subunits (Blednov et al., 2011) and those with complete *Gabra2* genetic deletion (Dixon et al., 2012). Together, these studies suggest that potentiating GABA_A_ receptors with α2 subunits, or the synapses from CCK-IN synapses that preferentially target their segments (Nusser et al., 1996; Nyiri et al., 2001), might confer some protection against the likelihood to develop AUD. Variation in *GABRG1* has also been linked with AUD (Covault et al., 2008; Enoch et al., 2009). Murine studies suggest that *Gabrg1* displays the restricted expression, again pointing to CCK-INs as a potential cellular target (Tasic et al., 2018). Associations between variation in *GABRA6* and *GABRG3* have also been made with alcohol dependence (Dick et al., 2004; Radel et al., 2005).

**Table 1:** Selected studies examining the association of GABA_A_ receptor gene single nucleotide polymorphisms (SNPs) with drinking or AUD vulnerability.

**Gene**	**Protein**	**Sample**	**Size**	**Finding**	**References**
*GABRA2*	α2 subunit	USA	446 dep / 334 non	Association with alcohol dependence	Covault et al., 2004
		COGA	2,282 dep / 1,254 non	Association with β frequency phenotype and alcohol dependence	Edenberg et al., 2004
		Japan	110 social drinkers	Association with subjective response to alcohol (0.05)	Roh et al., 2011
		USA	75 moderate drinkers	Association with happiness and vigor following alcohol (0.02 - 0.06)	Haughey et al., 2008
		USA	93 social drinkers	Association with stimulant properties of intoxication (0.10) in men but not women	Yang et al., 2017
*GABRA5*	α5 subunit	COGA	2,282 dep / 1,254 non	No associations	Dick et al., 2004
*GABRA6*	α6 subunit	Finland / Plains Indian	447 dep / 411 non	Association with alcohol dependence	Radel et al., 2005
*GABRB3*	β3 subunit	COGA	2,282 dep / 1,254 non	No associations	Dick et al., 2004
*GABRG1*	γ1 subunit	Finland / Plains Indian	447 dep / 411 non	Association with alcohol dependence	Enoch et al., 2009
		USA	132 moderate drinkers	No associations	Kosobud et al., 2015
*GABRG3*	γ3 subunit	COGA	2,282 dep / 1,254 non	Association with alcohol dependence	Dick et al., 2004

COGA, Collaborative Study on the Genetics of Alcoholism.

Genome-wide association studies (GWAS) have also been used to identify targets related to AUDs. A recent GWAS study (Meyers et al., 2017) identified *ZEB2*, a transcription factor that guides PV-IN and SST-IN migration (McKinsey et al., 2013), among several candidate genes underlying AUD. In addition, an association has been detected between AUDs and *SIX3* (Kranzler et al., 2019), another transcription factor important for neocortical development and interneuron maturation (Lagutin et al., 2003; Shi et al., 2021). In stark contrast to the litany of candidate gene studies, only two out of more than 20 contemporary genome-wide AUD association studies have identified a significant link with GABA_A_ receptors (Koulentaki and Kouroumalis, 2018). This inconsistency suggests a weak or modest link between GABA_A_ receptor function and AUD vulnerability. Nonetheless, the sheer volume of significant candidate gene studies, along with functional correlates, are consistent with a veritable association between *GABRA2* function and AUD vulnerability. The association between GABA_A_ receptor dysfunction and AUD is bolstered by imaging studies that revealed decreased benzodiazepine site availability across the cortex of patients with AUD (Abi-Dargham et al., 1998; Lingford-Hughes et al., 1998). The relatively weak effects observed in GWAS studies suggest that GABA receptor-related AUD phenotypes may be plastic and potentially overcome with the proper treatment. In addition, GWAS studies may not be sufficiently powered to detect genes from a highly comorbid and polygenic disease, such as AUDs. Nonetheless, while the genetic associations link inhibitory microcircuits with AUD diagnosis at the population level, the field is in need of post-mortem studies to investigate changes at the level of cytoarchitecture, protein expression, and/or transcript expression.

### Neurophysiology studies

Given that inhibitory INs are crucial for maintaining oscillatory activity, resting electroencephalography (EEG) studies can provide an instantaneous window into microcircuit function in patient populations. The EEG studies offer many technical and practical advantages over laborious neuroimaging techniques. One important caveat, however, is that signals can only be recorded from superficial structures, essentially limiting human studies to the neocortex. Continuous EEG recordings are commonly decomposed into the following frequency bands (in Hz): δ (<3), theta (4–7), α (8–12), β (13–29), and γ (>30) (Rangaswamy and Porjesz, 2014). Distinct INs contribute to oscillatory activity across these ranges (Kuki et al., 2015; Chen et al., 2017); therefore, disease-associated variation within a specific band may implicate one or more specific IN subtypes.

Changes to band activity following alcohol administration, and differences related to family or individual history, have been commonly observed within the α and β frequency bands (reviewed by Porjesz and Begleiter, 2003). Low-to-moderate doses of alcohol generally increase slow α power, particularly in the frontal cortices (Lukas et al., 1986, 1989; Ehlers et al., 1989; Ehlers and Schuckit, 1991; Cohen et al., 1993). Alcohol-induced increases in slow α power are exacerbated in individuals with a family history of AUD and correlate with the feeling of euphoria and desire to drink (Pollock et al., 1983; Lukas et al., 1986; Kaplan et al., 1988; Cohen et al., 1993). Alcohol-induced changes in the fast α spectrum, however, have shown conflicting results, possibly related to the effects of other medications or significant genetic stratification across self-identified racial groups (Ehlers and Schuckit, 1991). More consistently, alcohol administration also increases β frequency power, especially in at-risk individuals (Ehlers and Schuckit, 1990; Stenberg et al., 1994).

In the absence of alcohol, AUD diagnosis is associated with decreased α power (Jones and Holmes, 1976; Coutin-Churchman et al., 2006), and this effect is more pronounced in patients who have relapsed (Saletu-Zyhlarz et al., 2004). Decreased α power has also been observed in individuals with a family history of AUD (Propping et al., 1981; Ehlers and Schuckit, 1991). In contrast, β power, which is increased by alcohol itself, is also elevated in moderate drinkers and abstinent patients with AUD (Ehlers and Schuckit, 1990; Stenberg et al., 1994; Costa and Bauer, 1997; Rangaswamy et al., 2002; Coutin-Churchman et al., 2006). In addition, increased β power is more pronounced in recovering patients who relapse (Saletu-Zyhlarz et al., 2004), and may be more predictive of relapse than either illness severity and depressive symptoms (Bauer, 2001). Furthermore, the β power phenotype in patients with AUD has been linked with genetic variation in a locus encompassing *GABRA2, GABRA4, and GABRB1* (Porjesz et al., 2002; Edenberg et al., 2004) and also *ZEB2*, a transcription factor that regulates IN migration (McKinsey et al., 2013; Meyers et al., 2017). Together, these findings suggest that differences in frequency band power, particularly within the β range, may be considered an AUD endophenotype and leveraged as biomarkers for investigational treatments (Salvatore Curr Addict Rep 2015). EEG phenotypes have been observed in treatment-naïve and unmedicated patients (Bauer, 2001; Fein and Allen, 2005), but it is important to note that benzodiazepines and other medications may contribute to effects in broad patient populations with AUD. Another major caveat to neurophysiology studies in AUD has excluded female subjects so it is unclear how well these findings extend to all individuals. Thus, it is paramount that future studies assess sex as a biological variable.

While the consistent associations between EEG phenotypes and AUD diagnosis are striking, our mechanistic understanding of the IN populations underlying cortical EEG signatures remains limited. In adult mice, neocortical SST-IN activity can promote oscillations in the β frequency range (Chen et al., 2017). In contrast, PV-IN activity can suppress β power (Cho et al., 2015; Kuki et al., 2015) even though it promotes γ frequency activity (Gonzalez-Burgos et al., 2015). These findings suggest that SST-IN hyperactivity or PV-IN hypoactivity underlies increased β power and vulnerability to relapse in patients with abstinent AUD. For the moment, however, this hypothesis remains widely speculative without further preclinical studies. A relatively limited understanding of the basic microcircuits underpinning EEG power bands highlights the need for translational preclinical studies to provide context for clinical findings and to develop new testable hypotheses for treatment and biomarker development.

## Mechanistic insight from preclinical studies

### Ethanol modulates ionotropic GABA receptors

Due to its low molecular weight, hydrophilicity, and neutral charge, ethanol is readily distributed throughout the entire body and within all subcellular regions that contain water. These physiochemical properties confer ethanol with the ability to interact with a myriad of biological molecules. The use of stringent criteria regarding concentration-dependence, binding-site inhabitation, and genetic manipulation was recently suggested to define targets that underlie ethanol's anxiolytic, amnestic, sedative, and addictive properties (Abrahao et al., 2017). This literature will not be discussed here in detail, as ethanol's molecular targets have been reviewed by others (Harris et al., 2008; Trudell et al., 2014; Abrahao et al., 2017). While several specific findings have not been consistently replicated across laboratories, significant evidence supports the notion that ethanol induces adaptive behavioral effects by modulating fast neurotransmission within inhibitory microcircuits.

Ethanol modulates ionotropic receptors across the entire Cys-loop superfamily (Trudell et al., 2014), including glycine receptors (Perkins et al., 2010; Badanich et al., 2013), nicotinic receptors (Hendrickson et al., 2013; Gao et al., 2019), 5HT3a receptors (Parker et al., 1996; Lovinger, 1999), and, notoriously, GABA_A_ receptors. The acute actions of ethanol on the biophysical properties of isolated GABA_A_ receptors have been reviewed by others (Criswell and Breese, 2005; Weiner and Valenzuela, 2006; Lovinger and Homanics, 2007; Lobo and Harris, 2008). To describe this literature as controversial is a considerable understatement. Studies from isolated neurons have concluded that ethanol potentiates, does not affect, or inhibits currents elicited with exogenous GABA. In contrast, synaptic GABA_A_ receptor currents are more consistently facilitated by ethanol, although many studies have implicated an increased GABA release and not a direct action with the GABA_A_ receptor. Experiments conducted using recombinant expression systems raise additional questions about the physiological relevance of the direct interactions with GABA_A_ receptors, as ethanol is generally inefficacious at concentrations below 50 mM (~0.24 mg/dl). One noteworthy exception lies within a subset of oocyte studies suggesting that physiologically relevant concentrations of ethanol potentiate the conductance of GABA_A_ receptors containing α4, α6, and δ subunits (Sundstrom-Poromaa et al., 2002; Wallner et al., 2003; Wei et al., 2004; Bowen et al., 2015), but some conflicting results have also challenged whether this finding is widely reproducible (Borghese and Harris, 2007; Mehta et al., 2007). Despite the conflicting and inconclusive molecular literature, a litany of behavioral studies suggests that behavioral affects of ethanol occur through fast GABAergic transmission.

Convergent results from pharmacological and genetic studies indicate that interactions between ethanol and distinct pools of GABA_A_ receptors are relevant for behavioral effects and disease outcomes. Ethanol shares behavioral effects with many compounds that positively modulate GABA_A_ receptors, and conversely, GABA_A_ receptor negative modulators block these same effects (Grant, 1994; Criswell and Breese, 2005; Weiner and Valenzuela, 2006). Studies using transgenic mice also suggest that specific GABA_A_ receptor peptide residues are important for some behavioral effects of ethanol (Boehm et al., 2004; Kumar et al., 2009). Knock-in mice with ethanol-insensitive α1 subunits exhibit altered ethanol-induced anxiolysis and motor impairment (Werner et al., 2006). Similarly, the SNP in *Gabra6* confers α6-containing GABA_A_ receptors with enhanced sensitivity to ethanol and cerebellar motor impairing effects (Hanchar et al., 2005). In contrast, a similar mutation to α2 subunits does not affect anxiolysis or motor incoordination but alters volitional drinking, conditioned taste aversion, and ethanol's stimulant properties (Blednov et al., 2011; Newman et al., 2016). GABA_A_ α4 and δ subunits also regulate the discrete effects of ethanol, including binge drinking, motor effects, and abstinence-induced affective disturbances (Melon et al., 2018; Darnieder et al., 2019). The expression and function of these extrasynaptic subunits are modulated by neuroactive steroids and female sex hormones (Sundstrom-Poromaa et al., 2002; Stell et al., 2003; Maguire et al., 2005; Abramian et al., 2014), stressing the need to continue assessing sex as a biological variable. Finally, mice with an introduced point of mutation that enhances channel conductance in the GABA_A_ β1 subunit work harder to obtain ethanol and are more sensitive to intoxication (Anstee et al., 2013). These studies, along with many others not cited here, suggest that interactions between ethanol and specific GABA_A_ receptors mediate specific adaptive behavioral responses, consistent with ethanol acting across a constellation of distinct inhibitory synapses.

Considering that GABA_A_ receptor subunits vary with regard to their subcellular expression and inhibitory synapses in the mammalian forebrain arise from diverse groups of local GABAergic INs, the above findings suggest that distinct inhibitory microcircuits regulate the specific aspects of anxiolytic, sedative, and addictive properties of ethanol. The conclusion that ethanol exerts differential effects across GABA_A_ receptor subpopulations *in vivo*, in the context of the consistently conflicting findings from reduced systems, underscores strong motivation to understand how acute and chronic ethanol modulates heterogeneous inhibitory microcircuits. Examining how ethanol alters the physiology and circuit function of defined IN subpopulations should help coalesce existing gaps within the preclinical literature and uncover differences in the cellular substrates that mediate anxiolytic, sedative, and addictive properties of ethanol.

### Mechanisms of PFC GABAergic IN dysfunction in preclinical models

#### Acute ethanol administration

Studies examining how acute ethanol modulates neocortical GABA_A_ receptor function have yielded mixed results. In response to exogenous GABA, ethanol facilitates hyperpolarizing currents in neocortical neurons in culture (Aguayo, 1990; Reynolds and Prasad, 1991), acute slices (Proctor et al., 1992; Soldo et al., 1998), and intact systems (Nestoros, 1980). In contrast, PFC synaptic GABA_A_ receptor currents are unaffected by modest concentrations of ethanol (<50 mM) (Proctor et al., 1992; Marszalec et al., 1998; Criswell and Breese, 2005; Weitlauf and Woodward, 2008; Fleming et al., 2009). One potential mechanism contributing to this discrepancy is that ethanol may preferentially enhance extrasynaptic GABA_A_ receptors. Indeed, acute ethanol can induce or modulate tonic currents in pyramidal cells within the prelimbic PFC and the orbitofrontal cortex (Carlson et al., 2016a; Centanni et al., 2017). These tonic currents are generally mediated by α5- and δ-containing GABA_A_ receptors in the neocortex, but studies from the hippocampus have also suggested that ethanol can interact with α1- and δ-containing GABA_A_ receptors (Glykys et al., 2007). In addition, studies in the orbitofrontal cortex have shown that ethanol decreases current-evoked spiking by modulating glycine receptors (Badanich et al., 2013; Nimitvilai et al., 2016). Glycine receptors are also present and active in the prelimbic PFC (Salling and Harrison, 2014), but whether acute ethanol modulates them in the subregion has not been reported. Taken together, these studies indicate that under some conditions, acute ethanol can enhance inhibitory currents in the frontal cortex. Changes to α5-mediated tonic currents, potentially driven by SST-IN GABA release, may therefore underlie ethanol-induced changes to decision-making and cognition. Future studies using cell type-specific optogenetics are warranted to test this hypothesis with exciting ramifications for treatment development.

Several lines of evidence indicate that systemic ethanol delivery modulates neocortical inhibitory microcircuits. Low-to-moderate doses of ethanol (0.375–1.5 g/kg) decrease the mean firing rate of PFC pyramidal cells in rats (Tu et al., 2007) while other studies have shown that ethanol (1 g/kg) acutely decreases PFC GABA levels (Carton et al., 2019). While these findings are seemingly in opposition, a potential explanation is that ethanol may disrupt pyramidal cell synchrony by impairing PV-IN function. Accordingly, 25–50 mM ethanol (comparable to maximum concentrations reached by 1–3 g/kg) disrupts PV-IN up-state activity in slice culture (Woodward and Pava, 2009). In addition to the effects on PV-INs, a recent *in vivo* mouse study found that low doses of ethanol (0.5–1 g/kg) increase calcium mobilization in SST-INs while high doses (2–3 g/kg) decrease calcium activity (Li et al., 2021). These bidirectional changes in SST-IN calcium activity were paralleled by complementary changes in pyramidal cell activity. Furthermore, high doses of ethanol (3.5–5 g/kg) increase α4 localization in the synaptic compartment of pyramidal cells (Liang et al., 2007; Kumar et al., 2009; Carlson et al., 2014; Bohnsack et al., 2018). Together, these findings suggest that decreased SST-IN activity and increased pyramidal cell activity may trigger a homeostatic increase in GABA_A_ receptor function following ethanol exposure. Studies in slice culture have illustrated that these changes are dynamic, as α4 and δ subunit expression decrease following longer durations of ethanol exposure (Carlson et al., 2016b). These biphasic adaptations provide a potential mechanism contributing to acute ethanol tolerance (Liang et al., 2007; Gonzalez et al., 2012) and may have contributed to the significant variation observed in earlier studies.

Additional insight into the actions of ethanol on discrete IN types might be gleaned from studies in the hippocampus. In CA1 pyramidal cells, synaptic IPSCs evoked near the soma display enhanced sensitivity to ethanol (Weiner et al., 1997), raising the possibility that perisomatic PV-IN or CCK-IN synapses onto pyramidal cells may be especially sensitive to acute ethanol. On the other hand, spike-firing of spontaneously active INs located in the stratum lacunosum moleculare (potentially analogous to neocortical SST-INs and/or CCK-INs) is facilitated by 10–30 mM ethanol (comparable to maximum concentrations reached by 0.5–1 g/kg) (Yan et al., 2009). Ethanol has little effect on the intrinsic properties of quiescent INs, but in active INs, ethanol activates the pacemaker HCN channel current and increases spontaneous firing. Additional experiments in spontaneously firing INs within the stratum oriens (akin to SST-INs) revealed that concentrations of ethanol as low as 3 mM (less than maximum concentrations reached by 0.5 g/kg) enhance action potential frequency, again by facilitating HCN channel function (Yan et al., 2010). In contrast, low concentrations of ethanol (5–10 mM) may decrease the activity of some IN classes by attenuating kainate receptor transmission and induced spike-firing (Carta et al., 2003). Other functions of the kainate receptor were not disrupted, suggesting that substantial endogenous glutamate transmission is required for ethanol application to inhibit the IN activity. Thus, ethanol may preferentially exert some of its effects through inhibitory microcircuits with high glutamatergic tone. Collectively, these studies illustrate that acute ethanol induces heterogeneous responses to the synaptic and membrane physiology of distinct IN subclasses. In addition, the hippocampal and neocortical literature suggests that SST-INs may be the IN class with the greatest sensitivity to ethanol. Clearly, the presented compelling rationale supports revisiting effects of ethanol on the intrinsic properties of defined PFC INs using contemporary transgenic and optogenetic methodologies.

#### Moderate exposure and voluntary drinking

**Figure 2:**
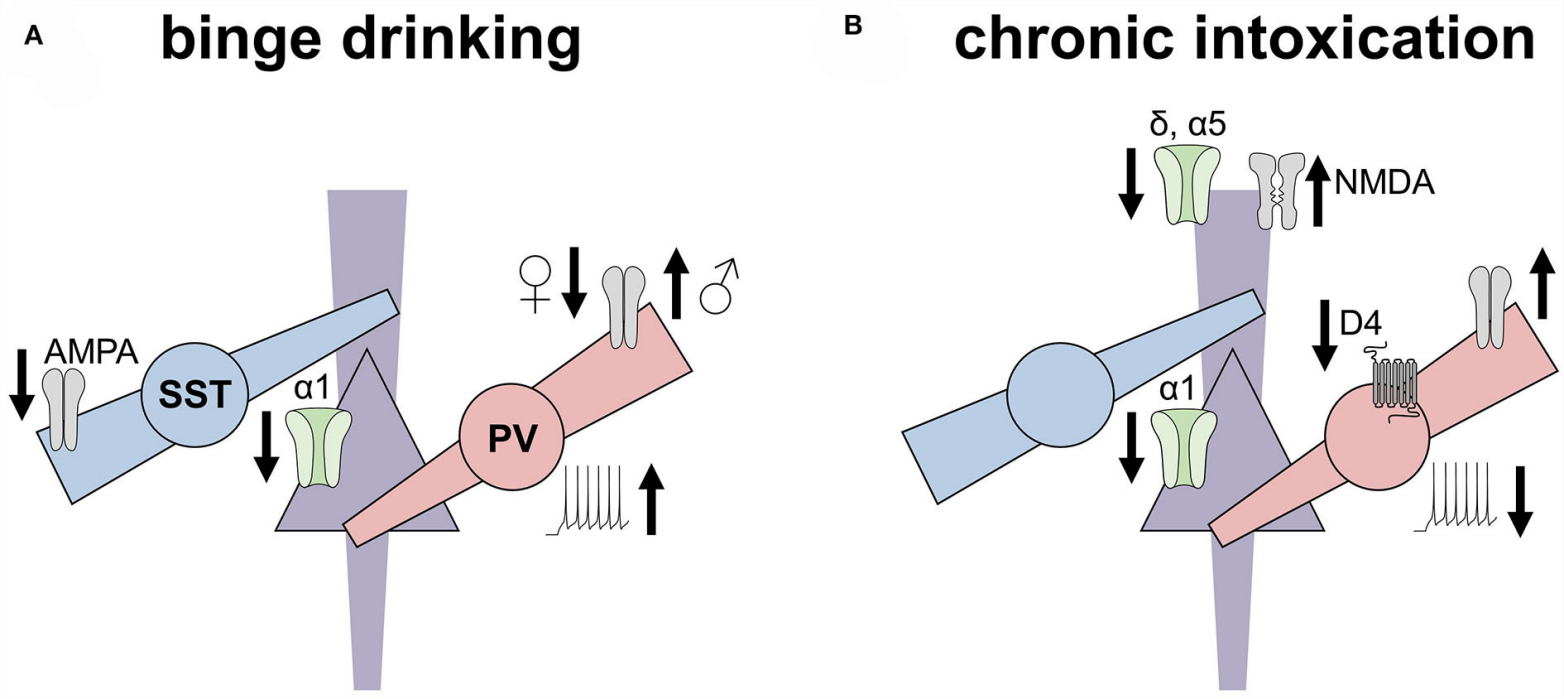
Ethanol experiences induced disparate adaptations to inhibitory microcircuits in the rodent prefrontal cortex. This non-exhaustive figure highlights key adaptations to the prefrontal cortex inhibitory microcircuits observed in rodent models of binge drinking (left) and dependence (right). **(A)** Excitatory drive onto somatostatin interneurons (SST-INs) is decreased following chronic voluntary drinking, along with a concomitant decrease in the expression of α1 GABA_A_ subunits on pyramidal cells. In contrast, excitatory drive onto parvalbumin (PV) INs is decreased in female mice but increased in male mice following binge drinking. PV-INs from drinking mice also exhibit enhanced excitability. **(B)** After chronic intoxication or dependence, however, PV-INs display reduced excitability relative to controls, along with reduced regulation by dopamine receptors. Increased excitatory drive onto PV-INs of both female and male mice has also been observed following the induction of ethanol place preference but not place aversion. Pyramidal cells from dependent animals display reduced α1 and α5 GABA receptor subunits, the latter of which may relate to increased NMDA receptor function observed in these models.

Adaptations to PFC microcircuitry have been observed in several binge drinking models (Figure 2A). Most rodent studies examining how voluntary drinking affects PFC inhibitory transmission have been conducted following intermittent two-bottle choice (2BC). In male rats, PFC *Gabra5* and *Gabrb1* expression each positively correlate with ethanol intake (Pickering et al., 2007), suggesting frontal cortex microcircuits, and tonic inhibition through α5 receptors may contribute to individual variation in the desire to drink. Consistent with this hypothesis, decreased *GABRA5* expression has been observed in the PFC of patients with AUD, an effect that is exacerbated in female individuals (Janeczek et al., 2020). Studies have also implicated changes in PFC phasic inhibitory transmission following binge drinking. Decreased *Gabra1* expression has been observed in the PFC of male rats that underwent intermittent 2BC (Bohnsack et al., 2018). Furthermore, Dao et al. (2021) recently found decreased sIPSC frequency following drinking in dark (DID) in mice, and 1-day abstinence from intermittent 2BC led to a trend decrease in sIPSC frequency and a prolonged sIPSC rise time in the pyramidal cells of male rats (Klenowski et al., 2016). These findings raise the possibility that drinking may alter the relative contributions of IN subpopulations to the inhibitory milieu (e.g., a decreased contribution of peri-somatic PV-IN activity to overall sIPSCs). Recently, Cannady et al. (2020) reported that 1-day abstinence from intermittent 2BC did not affect sIPSC amplitude or frequency in the anterior cingulate cortex of male mice. Kinetics parameters were not reported in these studies, precluding direct comparisons with some prior work in prelimbic PFC. Moreover, an important consideration for future experiments is that neocortical pyramidal cells can be differentiated into several subclasses based on projection target as well as the expression of GPCRs and other proteins. Thus, it is likely that disease-related experiences differentially alter the IN regulation of pyramidal cell subpopulations, as we have observed with respect to excitatory transmission (Joffe et al., 2021). Future studies will need to use *a priori* means to distinguish cell types to parse subtle or bidirectional changes to specific subpopulations. In addition, postmortem studies, in mixed-sex cohorts, should be conducted to assess whether changes in GABA_A_ receptor protein are apparent in patient populations.

Several lines of convergent evidence have related PFC PV-IN function with binge drinking. One-day abstinence from intermittent 2BC increases Fos expression in one-third of PFC INs in male rats (George et al., 2012), but it is unclear from those studies which class(es) of INs were involved. Contemporary studies leveraging transgenic tools are beginning to assess how ethanol experiences alter defined subtypes of PFC INs. A day after intermittent 2BC, we found that prelimbic PV-INs displayed enhanced excitability in mice of all sexes (Joffe et al., 2020). However, we observed a notable sex difference with regard to synaptic adaptations. After intermittent 2BC, PV-INs displayed diminished excitatory drive in female mice but enhanced postsynaptic AMPA receptor function in male mice. These adaptations to PV-INs in male mice provide two compelling mechanisms for the increased Fos expression observed in the prior male rat studies (i.e., increased excitability and/or synaptic drive). In addition, others have shown that genetic disruption of synaptic transmission onto cortical INs decreases voluntary drinking (Radke et al., 2017) and that PV-IN disinhibition increases binge drinking in male mice, but not female mice (Melon et al., 2018). Together, these studies support the hypothesis that increased activity of forebrain PV-INs contributes to drinking behaviors and that PFC PV-INs are involved in sex differences in top-down control.

Recent studies have begun to assess how drinking experiences modulate SST-IN physiology. In contrast to the findings from PV-INs, studies from our lab examining deep layer prelimbic SST-IN intrinsic properties in female mice or male mice found no differences associated with 1-day abstinence from intermittent 2BC (Joffe et al., 2020). Interestingly, in prelimbic layers 2/3, Dao et al. (2021) observed that one-day abstinence from DID was associated with decreased excitability of SST-INs. Together, these data suggest that there may be layer-specific or light phase-specific adaptations to SST-IN during early abstinence from voluntary drinking. An earlier study by Dao et. al (2020) examined SST-IN physiology following extended withdrawal from continuous 2BC in female mice, finding that SST-INs in the abstinence group exhibited *enhanced* current-evoked spike firing, a finding directly opposite to their studies conducted in early abstinence (Dao et al., 2021). The discrepancy between these two recent studies is likely to be stemmed from differences in the length of abstinence or between intermittent vs. continuous ethanol exposure. Another important consideration is that the enhanced SST-IN excitability was observed in slices from mice subjected to a battery of depressive-like behaviors, including a forced swim test 90 min before tissue collection (Dao et al., 2020). Thus, abstinence from ethanol may prime SST-INs for intrinsic excitability plasticity following acute stress. Testing whether these adaptations mediate stress-induced relapse will be an important series of future studies. Considering PFC SST-INs have been widely implicated in MDD etiology (Rajkowska, 2000; Tripp et al., 2011), adaptations to stressful stimuli (Xu et al., 2019; Cummings and Clem, 2020; Joffe et al., 2022), and rapid antidepressant action (Gerhard et al., 2019; Ali et al., 2020), the SST-IN system represents an exciting target for ameliorating the negative affective symptoms related to AUD (Robinson and Thiele, 2020; Crowley and Joffe, 2022).

Convergent findings suggest that decreased excitatory drive onto SST-INs emerges in early abstinence from voluntary drinking (Joffe et al., 2020; Dao et al., 2021). We also found a subtle sex difference in deep layer SST-INs, as electrically evoked EPSCs from male mice, but not female mice, displayed a decreased paired-pulse ratio following intermittent 2BC (Joffe et al., 2020). One potential explanation underlying this unexpected adaptation is that the paired-pulse ratio in SST-INs may reflect postsynaptic features, such as interactions with transmembrane AMPA receptor regulatory proteins or the intracellular polyamine block of CP-AMPA receptors (Rozov and Burnashev, 1999; Soto et al., 2007). Intriguing support for that hypothesis lies in a recent study that discovered female mice express more *Gria2* in PFC SST-INs than male mice (Gerhard et al., 2019). Thus, excitatory synapses onto SST-INs in female mice may not express CP-AMPA receptors to a greater degree, and receptor internalization following intermittent 2BC would not be expected to alter polyamine sensitivity. Future studies should explicitly examine AMPA receptor stoichiometry in SST-INs in all sexes, as these changes are likely to alter synaptic plasticity and the response to many potential SST-IN-directed treatments (Tomita et al., 2006). Sex differences in SST-IN-mediated disinhibition of other interneurons also appear to manifest following binge drinking, specifically in male individuals (Dao et al., 2021).

Despite the compelling molecular evidence indicating ethanol modulates 5HT_3_ receptor function (Lovinger and White, 1991; Lovinger, 1999), few studies have directly addressed how ethanol affects 5HT3-INs. The PFC transcript expression for the 5HT_3_ receptor (*Htr3a*) positively correlates with alcohol consumption in male rats (Pickering et al., 2007). In addition, the prefrontal cortex *Htr3a* expression positively correlates with reward learning following ethanol (0.5 g/kg) place conditioning (Xu et al., 2012). To the best of our knowledge, however, no studies have been published that directly assess whether acute exposure, voluntary drinking, or ethanol dependence alter the physiology of any subtype of PFC 5HT3-IN. Several studies, however, do implicate neuropeptides potentially released from PFC 5HT3-INs in the regulation of voluntary drinking. For instance, the site-specific delivery of NPY receptor or CRF receptor ligands can modulate binge drinking in the DID mouse model (Robinson et al., 2019a,b). Ethanol-naïve alcohol preferring P rats also exhibit lower levels of NPY immunoreactivity in the frontal cortex than controls, although NPY expression was not altered by ethanol exposure (Ehlers et al., 1998). Taken together, these studies provide compelling evidence for future studies to assess physiological adaptations across several genetically defined PFC IN subtypes in preclinical models of AUD. Discovering pharmacological agents to dampen PV-IN activity and/or boost excitatory drive onto SST-INs appear to provide particularly promising avenues toward attenuating symptoms associated with early abstinence.

#### High exposure and dependence

Several rodent studies have shown that high ethanol exposure differentially modulates inhibitory microcircuits across the frontal cortex areas (Figure 2B). An important caveat to this literature is that nearly all published PFC experiments in dependence models have been restricted to male subjects. Acute withdrawal from chronic intermittent ethanol (CIE), vaporized ethanol exposure for 16 h/day, 4 days/week, 1–4 weeks, is associated with decreased sIPSC frequency on pyramidal cells in the mouse infralimbic cortex (Pleil et al., 2015). Tetrodotoxin blocked this effect, indicating that early withdrawal disrupts active inhibitory microcircuits. Studies on the prelimbic cortex have yielded more conflicting results. In mice, acute withdrawal from CIE did not affect sIPSC parameters in studies from two laboratories (Trantham-Davidson et al., 2014; Pleil et al., 2015). On the other hand, acute withdrawal from chronic ethanol gavage decreased sIPSC frequency in the prelimbic cortex pyramidal cells in rats (Bohnsack et al., 2018; Hughes et al., 2019), and these adaptations have been corroborated by transcript and protein level changes (Devaud et al., 1997; Kumar et al., 2009; Bohnsack et al., 2018). The discrepancies in findings related to dependence effects on the prelimbic pyramidal cell function might be related to the route of ethanol administration and/or species; however, it is also possible that variation in the source of GABA during spontaneous recordings might have contributed to discrepancies across laboratories. Future studies using transgenic technologies to isolate outputs from specific IN classes will be important to reconcile discrepancies in the preclinical literature.

Studies directly targeting PFC INs suggest that ethanol dependence alters their physiology in a cell-autonomous manner. Trantham-Davidson and Chandler (2015) found that dopamine lost the ability to modulate PV-IN membrane physiology 1 week after withdrawal from CIE in male rats. In contrast, mGlu_1_ receptor potentiation retained the ability to modulate PV-INs, suggesting that CIE withdrawal precipitates differential changes to GPCR signaling cascades within INs. More recently, Hughes et al. (2020) used a viral-assisted approach to label INs in the rat PFC *via* the mDlx promotor (Dimidschstein et al., 2016). After 1 day of discontinuation from chronic ethanol gavage, INs were patched and functionally classified as “Fast-Spiking” (putative PV-INs) or “Martinotti” (putative SST-INs). Decreased PV-IN excitability was observed in cells from female and male ethanol-treated rats (Hughes et al., 2020). Similarly, we observed decreased excitability in fluorescently-identified PV-INs from transgenic mice following conditioned place preference to repeated ethanol injections (Ferranti et al., 2022). Interestingly, despite receiving identical ethanol exposure, PV-INs from mice that underwent conditioned place aversion were not different from controls, indicating that adaptations to PV-INs vary based on the learned experience. Based on these studies, manipulations that enhance PV-IN function may have the potential to remediate long-term physiological and behavioral changes associated with dependence. Additional studies need to be performed to better understand SST-IN and 5HT3-IN adaptations in models of high ethanol exposure.

#### Adolescent exposure

Across mammalian species, the PFC is one of the latest brain areas to fully mature, with the refinement of synaptic connections and improved cognitive functions not occurring until young adulthood (Kolk and Rakic, 2022). Understanding how drinking affects the adolescent brain is essential for modeling facets of AUD, as adolescents are more likely to binge drink than adults, and the early onset of drinking can increase the likelihood of an individual developing an AUD later in life (Hingson et al., 2006; Marshall, 2014).

Importantly, in preclinical studies, ethanol dependence during adolescence differentially affects PFC function relative to ethanol administered to adults (Trantham-Davidson et al., 2014, 2017; Barker et al., 2017). PV-INs in adult male rats exposed to 4 weeks of CIE during adolescence display decreased current-evoked firing relative to air controls (Trantham-Davidson et al., 2017). In addition, adolescent CIE decreases the sIPSC amplitude in adults and prevents the development of δ subunit tonic currents in male rats and female rats (Centanni et al., 2017). These effects were specific to deep layers, suggesting greater disruptions at synapses from PV-INs or SST-INs relative to 5HT3-INs. Results from several studies suggest that the tonic GABA_A_ receptor current in PFC pyramidal cells is developmentally regulated. Minimal tonic current has been reported in the frontal cortex of relatively young rats and mice (3–6 weeks) (Drasbek and Jensen, 2006; Weitlauf and Woodward, 2008; Salling and Harrison, 2014; Centanni et al., 2017), whereas PFC pyramidal cells from adult animals (>8 weeks) display tonic currents mediated by receptors containing α5 and/or δ subunits (Lee and Maguire, 2014; Centanni et al., 2017). These findings suggest that ethanol dependence during adolescence can stunt or delay PFC maturation (Spear and Swartzwelder, 2014; Centanni et al., 2017). Consistent with this notion, adolescent 2BC disrupts the developmental trajectory of PFC pyramidal cell intrinsic properties (Salling et al., 2018). In the hippocampus, as well, decreased δ and α4 GABA_A_ subunits have been observed following extended withdrawal from CIE delivery to adolescent male mice (Fleming et al., 2013; Centanni et al., 2014).

## Conclusion and future directions

In this review, we outlined the basic, translational, and population-level studies that implicate PFC inhibitory microcircuit function in the acute effects of ethanol and the symptoms and development of AUDs. Significant research has described the effects of ethanol on isolated components of IN synaptic transmission, and there are convergent findings between preclinical research and human studies using postmortem samples and genetics. Nonetheless, we are just beginning to understand how long-term ethanol exposure generates adaptations to defined IN classes and cell types. Emerging preclinical findings suggest that PV-IN intrinsic properties display biphasic adaptations, such that modest ethanol exposure increases but prolonged the exposure and decreases the dependence of PV-IN excitability. In addition, excitatory drive onto PV-INs may represent an exciting potential substrate underlying sex differences in alcohol-seeking and AUD vulnerability. In contrast, decreased excitatory drive onto SST-INs has been observed in multiple laboratories in mouse studies using all sexes, but it remains less clear how SST-IN physiology may be altered in models of high ethanol exposure.

Recent advances in transgenic tools, fluorescent biosensors, and opto-/chemogenetics provide unprecedented opportunities to assess ethanol-induced adaptations to discrete cell types and circuits, paving the way for the development of new rationally designed treatments for AUDs. Nonetheless, translating molecular and circuit-specific discoveries into new life-saving therapies persists as a fundamental challenge in neuroscience. What realistic interventions can target discrete cortical microcircuits in patients? In 30 years, perhaps, cell type-specific expression of optogenetic proteins, chemogenetic actuators, or other exogenous bioactive molecules may become viable treatment options for psychiatric diseases. Indeed, continued work identifying and optimizing viral promotors that enable cell type-restricted expression following systemic delivery (Dimidschstein et al., 2016; Vormstein-Schneider et al., 2020) holds a great promise for minimizing the invasiveness of approaches that require gene therapy. In addition, pharmacogenetic systems, like drugs acutely restricted by tethering (Shields et al., 2017), may provide opportunities to modulate any number of endogenous receptors on discrete cell populations.

Despite the promise of these next-generation approaches, such treatments will offer no relief for individuals suffering from AUDs within the next decade. At this time, brain stimulation and conventional pharmacology approaches remain the only safe and readily deployable ways to modulate brain function in the clinic. Due to its fortuitous location near the surface of the skull, the PFC is amenable to stimulation-based treatments including transcranial magnetic stimulation and transcranial direct current stimulation (Salling and Martinez, 2016; Philip et al., 2020). In recent years, several exciting and well-controlled trials have demonstrated that focal PFC stimulation can attenuate changes in the corticostriatal function observed in AUD populations and can alleviate cravings during abstinence (Hanlon et al., 2018). These groundbreaking studies have demonstrated exciting proof-of-concept that targeting PFC function can provide new therapies for AUDs. While the cells and mechanisms through which PFC brain stimulation can reduce alcohol cravings remain unresolved, we propose that cortical INs represent compelling candidates. TMS modifies neural circuits through repetitive rhythmic stimulations thought to entrain oscillatory activities (Lin et al., 2021). In addition, the long-term efficacy of brain stimulation approaches is thought to be related to persistent effects on synaptic plasticity (Hanlon et al., 2018). As discussed in this review, INs play key roles in regulating the endogenous processes recruited by TMS; therefore, we believe modulating PFC IN activities may provide synergistic opportunities to enhance the efficacy of brain stimulation approaches. Future mechanistic studies assessing the physiological adaptations that occur to define IN subtypes following brain stimulation would provide a promising avenue toward treatment optimization and may help identify new paths to treatment altogether.

Conventional pharmacology offers alternative means of targeting cortical microcircuits in the clinic. GABA_A_ receptor modulators have been used for decades to treat symptoms of acute alcohol withdrawal, anxiety, and sleep disturbances. Despite their utility and efficacy, these drugs are hampered by concerning side effects and their own liabilities for dependence. In general, drugs that minimize direct receptor agonism and that target more specific subunit combinations of GABA_A_ receptors exhibit safer clinical profiles; however, improvements are still very much possible and desired from GABA_A_ modulators currently in widespread use. Excitingly, the neuroactive steroid, brexanolone, a medication that works by potentiating δ-containing GABA_A_ receptors (Walton and Maguire, 2019), was recently approved for postpartum depression and deserves some attention for the potential to craft a microcircuit-based treatment for AUDs. To this end, we believe continued mechanistic studies examining PFC INs are essential. Because IN subtypes display specialized transcriptomes relative to other cell types, precise efforts to identify receptors selectively expressed by INs may yield novel druggable targets with circuit-specific actions. For example, the M2 subtype of muscarinic receptor displays restricted expression within a subclass of SST-IN and other types of INs (Hajos et al., 1998; McDonald and Mascagni, 2011), and variation in the M2 gene *CHRM2* predisposes individuals to AUD and is linked with the severity of illness (Wang et al., 2004; Jung et al., 2011). Based on this, the development of small molecule modulators of M2 receptors may represent a path to leverage endogenous neurobiological specificity toward circuit-specific therapies. The metabotropic glutamate receptor, subtype 1 represents another exemplar receptor whose preferential expression in SST-INs has the potential to be leveraged to develop microcircuit-specific pharmacological treatments (van Hooft et al., 2000; Maksymetz et al., 2021). In addition to receptors expressed by INs themselves, approaches that manipulate their targets may prove advantageous. Toward this goal, a variety of neuropeptide receptor systems (e.g., SST, VIP, CCK, and NPY) represent identified targets and relatively low-hanging fruit. Metabotropic and ionotropic receptors for GABA itself may also be targeted to generate circuit-specific treatments, although their relatively widespread expression, combined with the known side effect liability of existing GABAergic medications, seems to indicate that a pharmacogenetic approach may be required to selectively target these molecules.

Continued transcriptomics studies, in postmortem tissues and animal models, will be essential to identify endogenous molecules to target medication development. Now, more than ever, it is essential that parallel multiplex protein and *in situ* hybridization studies in human postmortem brain tissue should be conducted to assess whether phenomena described in rodents also occur in patient populations. Over the last 10 years, methodological advances (Sweet et al., 2010; Glausier et al., 2014, 2021; Fish et al., 2018) and the establishment of the NIH NeuroBioBank have created an incredible opportunity to learn more about AUDs by studying postmortem tissue. Floyd Bloom said, “The gains in brain are mainly in the stain.” Similar to the new James Webb Space Telescope which is going to allow scientists to explore uncharted galaxies, blackholes, and planetary systems, the ability to perform quantitative, highly multiplexed immunofluorescence imaging of human postmortem tissue from subjects with AUDs will undoubtedly provide new insights to the etiology and provide valuable information about potential drug targets. For example, quantitative fluorescence microscopy was recently used to show that the density of synapses arising from a unique chandelier GABA neuron subtype is higher in PFC layer 2 of subjects with schizophrenia relative to matched comparison subjects (Rocco et al., 2017). Moreover, studies of postmortem tissue are not limited to just immunohistochemistry and immunocytochemistry: it is possible to perform multiplex RNA *in situ* hybridization (Fish et al., 2018), proteomics (MacDonald et al., 2019), and genomic (e.g., RNAseq) (Arion et al., 2007; Enwright and Lewis, 2021) analyses at once. Importantly, the methods used to collect tissue are becoming standardized such that high quality tissue is readily available (Stan et al., 2006). In addition, the techniques being employed can be used to rapidly assess large cohorts so that potentially confounding comorbidities can be assessed. Rigorously examining PFC microcircuit function in parallel studies in preclinical models and postmortem tissues holds a great promise for the development of breakthrough treatments for AUDs.

## Author contributions

MJ prepared the first draft. KF and MJ equally contributed to revising all subsequent drafts. Both authors contributed to the article and approved the submitted version.

## Funding

This study was supported by the NIH (K99/R00AA027806 to MJ and MH096985 to KF).

## Conflict of interest

The authors declare that the research was conducted in the absence of any commercial or financial relationships that could be construed as a potential conflict of interest.

## Publisher's note

All claims expressed in this article are solely those of the authors and do not necessarily represent those of their affiliated organizations, or those of the publisher, the editors and the reviewers. Any product that may be evaluated in this article, or claim that may be made by its manufacturer, is not guaranteed or endorsed by the publisher.
